# Memorial, by Dr. Henderson, Addressed to the Committee Who Undertook the Investigation of Ann Moore's Case
*At the time we stated, at page 79 of this Journal, our opinion of the observations made by Dr. Henderson in his pamphlet, being the means of bringing out a complete detection of Ann Moore, we did it on the publicity of the circumstance, not having then received any private intimation of a contrary fact. If we have been in an error in this particular, we acknowledge an obligation to Mr. Webster. Our great wish is to lay before the public as much of clear and incontrovertible fact as possible. As an opinion, however, our former assertion has not lost its weight, for we still think that the investigation and examination of Ann Moore's case by so acute an observer as Dr. Henderson, had a principal if not an exclusive share in the *denouement* at Tutbury. The above *Memorial* will show how much Dr. Henderson has interested himself on this subject, and also how far the Committee may have been indebted to him for the mode of conducting the watch.—Editors.


**Published:** 1813-09

**Authors:** 


*For the Medical and Physical Journal.*

### *MEMORIAL, *by* DR. HENDERSON, *addressed to the* COMMITTEE *who undertook the* INVESTIGATION *of* ANN MOORE'S CASE.

HAVING understood, that Ann Moore has, within these few days, given her consent to a repetition of the watching of her person, I would beg leave to call the attention

* At the time we stated, at page 79 of this Journal, our opinion of the observations made by Dr. Henderson in his pamphlet, being the means of bringing out a complete detection of Ann Moore, we did it on the publicity of the circumstance, not having then received any private intimation of a contrary fact. If we have been in an error in this particular, we acknowledge an obligation to Mr. Webster. Our great wish is to lay before the public as much of clear and incontrovertible fact as possible. As an opinion, however, our former assertion has not lost its weight, for we still think that the investigation and tion of the gentlemen who may undertake the task, to those precautions which, it appears to me, ought to be observed on the occasion.

In the first place, as it is evidently the interest of the inhabitants of Tutbury to encourage and support an imposition which attracts so many visitors to their village, I think that Ann Moore should be removed from Tutbury to some place in the neighbourhood, as, for instance, to Burton, where the chance of connivance and collusion would be less.

With regard to the gentlemen who may undertake the office of watching, it is of the utmost importance that they should be free from all suspicion of an undue bias. None of Ann Moore's private friends, no one who has had much previous communication with her, no one who has committed himself on the subject of her fasting, by writing, or otherwise, ought to be permitted to approach her person while the trial continues. The number of watchers should not exceed four, or at most six; and of these only two should attend at one time, except when any general communication is to be made. It would be desirable that half of the number should be medical men, of whom one should be constantly present, or within call. A regular and minute journal of their observations and proceedings must be kept. They may relieve one another every six or eight hours.

In conducting the watch, the following particulars ought to be attended to. The patient being removed to a proper situation, should be placed in a room that does not directly communicate with the street, as was the case on the last trial. The bed should be without curtains, and must be carefully examined, before the watching commences, and when it terminates. It would be well to have it so constructed, that she can be weighed, while lying in it. At all events her body ought to be weighed at the commencement, and at the end of the watching, and twice a-day during the continuance of it. No one, except the persons employed in conducting and superintending the watch, must be permitted to enter the room, or, if allowed to do so, a screen, or rail, ought to be placed so as to prevent them from approaching within a certain distance from the bed. No food ought, on any pretence, to be introduced into the room;

---

and examination of Ann Moore's case by so acute an observer as Dr. Henderson, had a principal if not an exclusive share in the *denouement* at Tutbury. The above *Memorial* will show how much Dr. Henderson has interested himself on this subject, and also how far the Committee may have been indebted to him for the mode of conducting the watch.—Editors.

and and if the patient require water for the purpose of washing her face, it should be allowed only once a-week, the time that she has herself specified as the period of such indulgence; and then the operation should be performed by another person, not by herself. Her pulse and respiration, and the temperature of her body, ought to be from time to time examined; and it should be ascertained whether she sleeps soundly, or only slumbers.

Other observations will doubtless suggest themselves to those who superintend the watching; and further precautions may be necessary: but any omission of, or material deviation from those which I have particularized, will render the proof afforded by a new watching very unsatisfactory and inconclusive. The artifices of impostors are endless, and the most observant may be sometimes deceived. One of the alleged fasters, to whose history I have adverted in my "Examination of Ann Moore," and who was watched in an apparently unexceptionable manner, at two different periods, was at length discovered to have supported herself by means of food concealed within the hem of her gown: and another carried on the imposture by means of a box made so as to resemble a bible, which served as her provision chest.

*G. S, April* 5, 1813. A. H.

